# Short- and long-term risk stratification in acutely ill medical patients by implementing D-dimer in the emergency setting – A prospective cohort study

**DOI:** 10.1016/j.ahjo.2026.100783

**Published:** 2026-04-13

**Authors:** Andrea Kornfehl, Roman Brock, Julia Oppenauer, Felix Eibensteiner, Christoph Veigl, Marco Neymayer, Karolina Valentova, Alexandra Julia Lipa, Markus Müller, Tim Dreier, Philip Verdonck, Patrick Mucher, Thomas Perkmann, Helmuth Haslacher, Oliver Schlager, Sebastian Schnaubelt

**Affiliations:** a Department of Emergency Medicine, Medical University of Vienna, Austria; b Intensive Care Unit 13i2, Department of Internal Medicine I, Medical University of Vienna, Austria; c Division of Angiology, Department of Medicine II, Medical University of Vienna, Austria; d Division of Haematology and Haemostaseology, Department of Internal Medicine I, Medical University of Vienna, Austria; e Department of Emergency Medicine, Antwerp University Hospital, Belgium; f Department of Laboratory Medicine, Medical University of Vienna, Austria; g Emergency Medical Service Vienna, Austria

**Keywords:** Emergency department, D-dimer, Risk prediction, Mortality

## Abstract

**Objective:**

D-dimer testing is commonly used in emergency departments (EDs) to rule out thromboembolic events. However, elevated D-dimer levels may also be associated with an increased risk of mortality, independent of thromboembolism.

**Methods:**

This prospective observational study enrolled acutely ill medical ED patients. D-dimer measurements were conducted as part of biobanking at ED presentation. Survival at 30, 90, and 360 days was analyzed in relation to D-dimer, and Receiver operating characteristic (ROC) curves and logistic regressions were performed.

**Results:**

A total of 1035 patients were included (56% male; 61 years [IQR 46–74]). Median follow-up was 764 days (7.7% mortality). Elevated D-dimer (≥0.5 μg/mL) was observed in 46.4% of patients. Thirty-day mortality was 4.8% in patients with elevated D-dimer versus 0.2% with normal D-dimer levels (log-rank *p* < 0.001), while 360-day mortality was 13.5% versus 2.3% (*p* < 0.001), respectively. Among discharged patients, 360-day mortality was 9.2% with elevated D-dimer vs. 0.7% with normal values (log-rank p < 0.001).

The ROC-derived optimal cut-off for both time points was 0.75 μg/mL (AUC 0.82 and 0.76 for 30- and 360-day mortality). Multivariable analysis adjusted for age, BMI, and comorbidities yielded elevated D-dimer to remain an independent predictor of mortality.

**Conclusion:**

In unselected ED patients, D-dimer independently predicts 30- and 360-day mortality. Beyond its diagnostic role for VTE, an elevated D-dimer—particularly in patients with no VTE and discharge from the ED—identifies individuals at increased short- and long-term risk. Incorporating D-dimer (e.g., ≥ 0.75 μg/mL) into routine risk stratification may support targeted follow-up and post-discharge management.

## Highlights

• D-dimer is commonly used to rule out thromboembolic disease.
• In this study, D-dimer predicted short and long-term mortality in all acutely ill patients.
• Therefore, D-dimer could help risk-stratify all emergency patients.

## Graphical abstract

**Figure:**
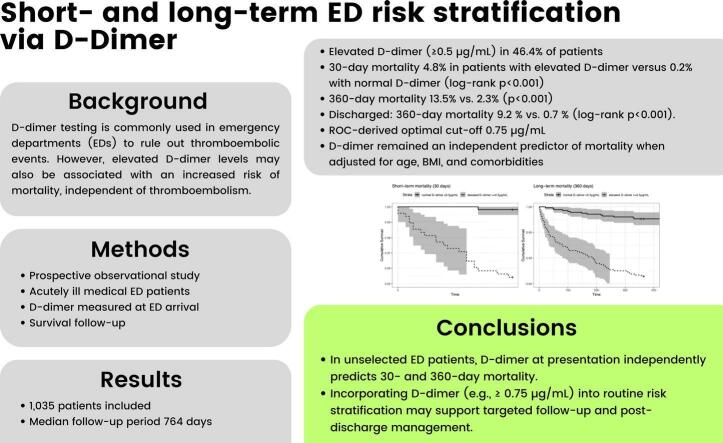

## 1 Background

D-dimer is a fibrin degradation product which is elevated in patients with acute venous thromboembolism (VTE), and is commonly utilized in emergency departments (ED) to rule out pulmonary embolism (PE) and deep vein thrombosis (DVT) as manifestations of VTE. [1], [2] Nevertheless, in daily ED practice, elevated D-dimer levels are frequently encountered even in patients without confirmed VTE, often leading to diagnostic uncertainty and overuse. [3], [4] Beyond VTE, elevated D-dimer levels reflect systemic activation of coagulation and fibrinolysis, which may be triggered by a wide range of conditions including infection, malignancy, trauma, cardiovascular disease, or generalized inflammation. [5], [6] While often dismissed as “false positives” in a narrow diagnostic sense, these elevations may, in fact, signal clinically relevant morbidity. [7], [8] Growing evidence suggests that elevated D-dimer levels may also serve as a marker of adverse outcomes, including all-cause mortality. [8], [9], [10], [11] Several cohort studies have found associations between elevated D-dimer levels on admission and increased short- and long-term mortality in various patient populations, for instance those with sepsis, heart failure, or respiratory failure including severe acute respiratory syndrome coronavirus 2 (SARS-CoV-2) infection, and even healthy adults. [12], [13], [14], [15], [16], [17], [18], [19], [20]

In the setting of the ED, especially under conditions of diagnostic uncertainty or limited resources, rapid and easily accessible markers of adverse outcomes are crucial for initial risk stratification and immediate clinical decision-making. Incorporating elevated D-dimer levels without a subsequent finding of VTE into general prognostic considerations could guide acute management strategies: identifying biomarkers that also provide prognostic information on long-term outcomes could optimize post-discharge planning, inform follow-up intensity, and potentially improve patient outcomes. Nevertheless, the usefulness of D-dimer as a prognostic marker for long-term outcomes in unselected ED populations is unclear. The aim of this study was therefore to investigate the prognostic value of D-dimer levels measured at ED presentation in relation to both short- and long-term mortality and to explore whether D-dimer may function as a generalizable biomarker for morbidity and mortality beyond its traditional use in VTE diagnostics.

## 2 Methods

### 2.1 Study design and patients

From November 2018 to April 2024, this prospective observational study included patients seeking care at the ED of the Medical University of Vienna, a tertiary academic hospital in Austria. The manifold reasons for presenting to the ED were classified into the categories chest pain, dyspnea, palpitations (e.g. atrial fibrillation or atrial flutter), suspected acute renal failure, and others (mainly infections). The study ED does not treat trauma or pediatric cases, so no trauma patients or children were screened or included. In addition, patients were not screened in case of hemodynamic or respiratory instability because measurements would have potentially resulted in a delay of necessary life-saving measures. Moreover, pregnant patients were not included, as D-dimers are known to be often elevated anyway. Thus, all patients aged over 18 presenting to the ED on days on which study personnel was present and who gave verbal and written informed consent were eligible for inclusion. General clinical information and patient demographics were noted at time of inclusion; other diagnostic and therapeutic information was collected from patient charts after hospital discharge. Follow-up regarding mortality was conducted via the governmental statistics office (Statistics Austria) after the enrolment phase of the biobanking study ended.

D-dimer (μg/mL) levels were measured directly after blood was drawn via the standard laboratory routine (citrate plasma, latex-agglutination). [21] If the treating physicians did not actively order a D-dimer for the patient at hand, the values were omitted from the result sheets by the laboratory department as part of the study. This way, no influence was possible on the standard diagnostic and therapeutic flow. An elevated D-dimer level was defined as being equal to or greater than 0.5 μg/mL or age-adjusted. [19], [21], [22]

This study was performed in accordance with good clinical practice guidelines and the declaration of Helsinki. The study was approved by the institutional Ethics Committee of the Medical University of Vienna, Austria (no. 2197/2017, approval 01/2018). Data reporting was performed according to STROBE guidelines.

### 2.2 Exposures and outcomes

The primary exposure of interest was plasma D-dimer concentration measured at patients' presentation to the ED. D-dimer was analyzed as both a continuous variable and a dichotomous variable, with the latter being categorized at a threshold of 0.5 μg/mL in line with established diagnostic practice. [21], [22] In secondary analyses, age-adjusted D-dimer cut-offs were applied. [19] Patients were also stratified according to discharge versus hospital admission status, and subgroup analyses were conducted by primary reason for ED presentation.

The primary outcome was all-cause mortality within 30 days of presentation to the ED. Secondary outcomes included all-cause mortality within 360 days and overall survival during the entire follow-up period. Cause of death was determined from clinical records, registry data, or information from the treating physician, and was classified as progression of the underlying disease, infection, or unknown. Exploratory subgroup outcomes were analyzed according to the main reason for ED presentation.

### 2.3 Statistical analysis

Continuous variables are presented as the median and IQR, and categorical variables are presented as absolute counts and percentages. Group comparisons were performed using the chi-squared test or Fisher's exact test for categorical variables, and the Mann–Whitney *U* test for continuous variables, as appropriate. Survival was analyzed using the Kaplan–Meier method, and differences between groups were compared using the log-rank test. Receiver operating characteristic (ROC) curves were generated to evaluate the predictive ability of D-dimer concentrations in predicting short- and long-term mortality. Area under the curve (AUC) values and 95% confidence intervals (CIs) are reported. Optimal cut-off values were determined by maximizing the Youden index. Additionally, univariate and multivariate logistic regression analyses were performed to explore the association between clinical variables and mortality at 30 and 360 days. The variables included in the multivariable models were selected based on their clinical relevance and statistical significance in the univariate analyses. Given the limited number of outcome events, the number of covariates was restricted to reduce the risk of overfitting. Odds ratios (OR) with 95% CIs are reported. All statistical tests were two-sided and a *p*-value of less than 0.05 was considered statistically significant. Missing data were handled using complete-case analysis. The analyses were conducted using R (version 4.3.1; R Foundation for Statistical Computing, Vienna, Austria).

## 3 Results

### 3.1 Study population characteristics

Between November 2018 and April 2024, 1035 acutely ill medical patients were enrolled. The median age was 61 (IQR 46–74) years, and patients were predominantly male (*n* = 577, 55.7%). The main reason for the ED visit was chest pain (*n* = 591, 57.1%), followed by dyspnea (*n* = 113, 10.9%) and suspicion of SARS-CoV-2 infection (*n* = 112, 10.8%). 8.1% (*n* = 84) of patients complained about palpitations with atrial fibrillation/atrial flutter as the underlying cause. Acute renal failure was suspected in 56 patients (5.4%). Seventy-nine (7.6%) presented to the ED for another reason, and this group mostly included suspected infections (except SARS-CoV-2).

### 3.2 D-dimer

A D-dimer test was carried out in 100% of included patients. The median D-dimer value was 0.45 μg/mL (IQR 0.27–1.05). The test result was elevated (≥0.5 μg/mL) in 480 patients (46.4%) and age-corrected elevated in 386 patients (37.3%). Of the 1035 patients who underwent D-dimer testing, 284 (27.4%) had their D-dimer values requested by their treating physicians as part of routine clinical practice. In these patients, the median D-dimer value was 0.59 μg/mL (IQR 0.34–1.51), elevated in 165 patients (58%) and age-corrected elevated in 142 patients (50%).

### 3.3 Mortality

**Fig. 1:**
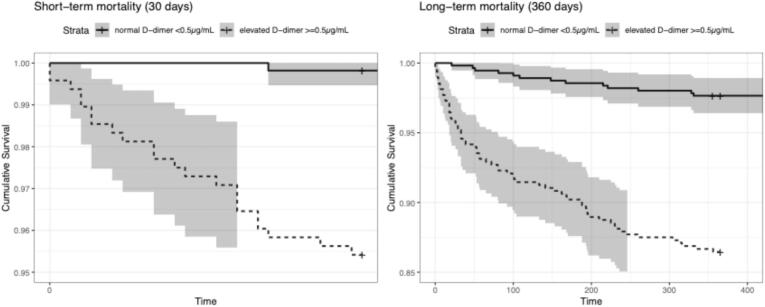
Kaplan Meier curve showing the cumulative probability of survival in acutely ill medical patients. The curves are categorized by their D-dimer value (normal [<0.5]/elevated [≥0.5μg/mL]).

**Fig. 2:**
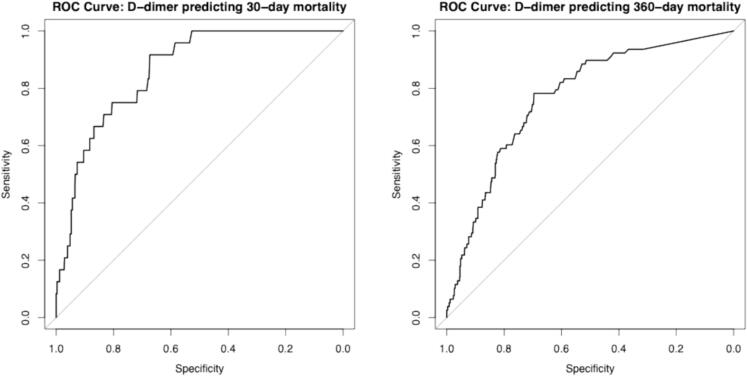
Receiver operating characteristic (ROC) curves for predicting short- (30-days) and long-term (360-days) mortality using D-dimer levels (≥0.5μg/mL).

The median survival time for the entire study population was 764.0 days (IQR 621.0–1078.0) until the cut-off time (study conclusion). Mortality within one year of ED presentation amounted to 7.7% of all patients (*n* = 80). Eight patients were lost to follow-up. The median age at death was 74.0 (IQR 63.5–80.5) years. Short-term (30-day) mortality was 4.8% (*n* = 23) in patients with an elevated D-dimer (age-corrected 4.9% [*n* = 19]), and 0.2% (n = 1; age-corrected 0.8% [*n* = 5]) in patients with normal D-dimer (log-rank *p* < 0.001; age-corrected p < 0.001). There was also a significant difference over the entire observational period regarding cumulative mortality *(*Fig. 1*)*: long-term (360-day) mortality was 13.5% (*n* = 65) in patients with an elevated D-dimer (age-corrected 15.3% [*n* = 59]), and 2.3% (*n* = 13; age-corrected 2.9% [*n* = 19]) in patients with normal D-dimer (log-rank *p* < 0.001; age-corrected p < 0.001) *(*Table 1*)*. Based on ROC analysis *(*Fig. 2*)*, the optimal cut-off for D-dimer for short-term mortality was 0.75 μg/mL, yielding the highest Youden index (J = 0.59), with a sensitivity of 91.7% and a specificity of 67.4%. For long-time mortality *(*Fig. 2*)*, the optimal cut-off for D-dimer was also 0.75 μg/mL, yielding the highest Youden index (J = 0.48), with a sensitivity of 77.5% and a specificity of 70.0%. A total of 56.3% of patients (*n* = 45; 4.3% of the entire cohort) died because of their underlying disease progressing (e.g., malignancy or severe coronary heart disease). Nine patients (11.3%; 0.9% of the entire cohort) died from an infection (e.g., SARS-CoV-2, bacterial pneumonia, or urinary tract infection). The cause of death could not be clearly determined in 32.6% of cases (*n* = 26; 2.5% of the entire cohort).

**Table 1:** Demographic, clinical characteristics, and mortality of 1035 acutely ill medical patients. IQR = interquartile range, St.p. = status post, μg/mL = μgramm/mL, SARS-CoV-2 = severe acute respiratory syndrome coronavirus 2.

		Overall (*n* = 1035)	D-dimer positive (*n* = 480)	D-dimer negative (*n* = 555)	p-value
Age (years)	median (IQR)	61.0 (46.0–74.0)	70.0 (57.0–79.0)	53.0 (41.0–67.0)	<0.001
Sex – female	n (%)	458 (44.3)	215 (44.8)	243 (43.8)	0.754
BMI (kg/m^2^)	median (IQR)	26.6 (23.5–30.2)	26.6 (23.4–30.0)	26.7 (23.7–30.4)	0.297
Reason for Presentation to the Emergency Department					
Chest pain	n (%)	591 (57.1)	216 (45.0)	375 (67.6)	<0.001
Dyspnoe	n (%)	113 (10.9)	76 (15.8)	37 (6.7)	<0.001
Palpitations/atrial fibrillation	n (%)	84 (8.1)	25 (5.2)	59 (10.6)	0.001
Suspected acute renal failure	n (%)	56 (5.4)	48 (10.0)	8 (1.4)	<0.001
Suspected SARS-CoV-2-infection	n (%)	112 (10.8)	72 (15.0)	40 (7.2)	<0.001
Other	n (%)	79 (7.6)	43 (9.0)	36 (6.5)	0.159
Comorbidities					
Arterial hypertension	n (%)	521 (50.3)	282 (58.8)	239 (43.1)	<0.001
Hyperlipidemia	n (%)	296 (28.6)	158 (32.9)	138 (24.9)	0.005
Diabetes mellitus (type II)	n (%)	186 (18.0)	108 (22.5)	78 (14.1)	<0.001
Chronic kidney disease	n (%)	91 (8.8)	67 (14.0)	24 (4.3)	<0.001
Peripheral arterial disease	n (%)	54 (5.2)	37 (7.7)	17 (3.1)	0.001
Cerebral arterial disease	n (%)	56 (5.4)	33 (6.9)	23 (4.1)	0.055
St.p. insult	n (%)	57 (5.5)	39 (8.1)	18 (3.2)	<0.001
Coronary heart disease	n (%)	199 (19.2)	112 (23.3)	87 (15.7)	0.002
St.p. myocardial infarction	n (%)	155 (15.0)	68 (14.2)	87 (15.7)	0.541
Nicotine abuse	n (%)	410 (39.6)	179 (37.3)	231 (41.6)	0.161
Laboratory values					
D-dimer (≥0.5 μg/mL)	median (IQR)	0.45 (0.27–1.05)	1.14 (0.73–2.31)	0.27 (0.27–0.35)	<0.001
Follow-up and Mortality					
Discharged from the ED	n (%)	646 (62.4)	240 (50.0)	406 (73.2)	<0.001
Admitted to the hospital	n (%)	389 (37.6)	240 (50.0)	149 (26.8)	<0.001
30 days	n (%)	24 (2.3)	23 (4.8)	13 (2.3)	<0.001
180 days	n (%)	41 (4.0)	37 (7.7)	4 (0.7)	<0.001
360 days	n (%)	78 (7.5)	65 (13.5)	1 (0.2)	<0.001

### 3.4 Follow-up

**Fig. 3:**
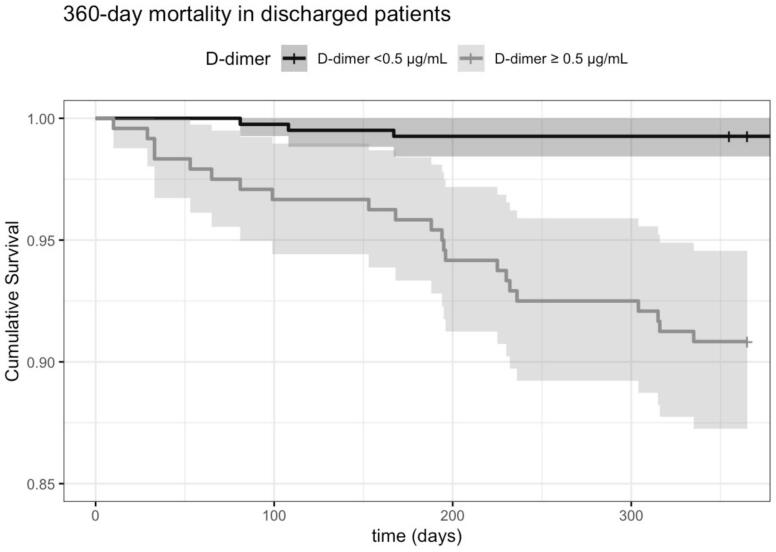
The Kaplan-Meier curve shows the cumulative probability of survival in acutely ill medical patients who were discharged after outpatient assessment. The curves are categorized by their D-dimer value (normal [<0.5 μg/mL] or elevated [≥0.5 μg/mL]).

Following ED proceedings, 62.4% (*n* = 646) of patients were discharged home. Of the D-dimer-positive patients, 50% were discharged, compared to 73.2% (*n* = 406) of the D-dimer-negative patients (*p* < 0.001). Long-term (360-day) mortality was 9.2% (*n* = 22) in discharged patients with elevated D-Dimer (age-corrected 11.1% [*n* = 21]) and 0.7% (*n* = 3; age-corrected 0.9% [n = 4] in discharged patients with normal D-dimer (log-rank p < 0.001; age-corrected p < 0.001) *(*Fig. 3*)*. For patients admitted to the hospital, long-term (360-day) mortality was 17.9% (*n* = 43; age-corrected 19% [*n* = 38]) with an elevated D-Dimer, and 6.7% (*n* = 10; age-corrected 15% [*n* = 15)]) with a normal D-Dimer value.

### 3.5 D-dimer as part of the clinical routine

The median survival time for patients for whom D-dimer was determined as part of the clinical routine was 892 days (IQR 586–1459). Short-term (30-day) mortality was 1.8% (*n* = 5) in patients with an elevated D-dimer and 0% (*n* = 0) in patients with normal D-dimer (log-rank *p* = 0.067). Long-time (360-day) mortality was 8.1% (*n* = 23) in patients with an elevated D-dimer and 0.0% (n = 0) in patients with normal D-dimer (log-rank *p* < 0.001).

### 3.6 Subgroups - primary reason ED presentation

In the subgroup analysis, the median D-dimer value was highest among patients who presented to the ED primarily due to dyspnea (0.82 [interquartile range (IQR) 0.36–2.16] μg/mL), followed by those with suspected SARS-CoV-2 infection (0.75 [IQR 0.40–1.60]). The proportion of positive D-dimer values was also highest in these two groups (dyspnea: 67.3%, *n* = 76; suspected SARS-CoV-2 infection: 64.3%, *n* = 72). The subgroup of patients who presented to the ED with suspected acute renal failure had the highest short-term (30 days; 12.5%, n = 7) and long-term mortality (360 days; 23.3%, *n* = 13). None of the patients in the palpitations/atrial fibrillation-, suspected acute renal failure-, dyspnea-, chest pain-, and negative D-dimer subgroups died within 30 days. Only one patient in the suspected SARS-CoV-2 infection subgroup with a negative D-dimer died within 30 days *(*Table 2*)*.

**Table 2:** D-dimer and mortality classified according to primary reason for presentation at the emergency department. μg/mL = μgramm/mL, IQR = interquartile range, positive = D-dimer ≥0.5μg/mL, negative ≤0.5μg/mL, SARS-CoV-2 = severe acute respiratory syndrome coronavirus 2.

		Suspected SARS-CoV-2 infection (n = 112)	Palpitations/atrial fibrillation (*n* = 84)	Suspected acute renal failure (*n* = 56)	Dyspnoe (n = 113)	Chest pain (n = 591)	p-Wert
D-dimer							
μg/mL	median (IQR)	0.75 (0.40–1.60)	0.32 (0.27–0.65)	1.30 (0.62–3.14)	0.82 (0.36–2.16)	0.36 (0.27–0.70)	
positive	n (%)	72 (64.3)	25 (29.8)	48 (85.7)	76 (67.3)	216 (36.5)	<0.001
negative	n (%)	40 (35.7)	59 (70.2)	8 (14.3)	37 (32.7)	375 (63.5)	<0.001
Mortality							
30 days	n (%)	6 (5.4)	0 (0.0)	7 (12.5)	2 (1.8)	5 (0.8)	<0.001
360 days	n (%)	12 (10.7)	8 (9.5)	13 (23.3)	12 (10.6)	25 (4.2)	<0.001
Mortality – D-dimer positive							
		n = 72	*n* = 25	n = 48	n = 76	*n* = 216	
30 days	n (%)	5 (6.9)	0 (0.0)	7 (14.6)	2 (2.6)	5 (2.3)	0.003
360 days	n (%)	10 (13.9)	6 (24.0)	13 (27.1)	10 (13.2)	19 (8.8)	0.007
Mortality – D-dimer negative							
		n = 40	n = 59	n = 8	*n* = 37	*n* = 375	
30 days	n (%)	1 (2.5)	0 (0.0)	0 (0.0)	0 (0.0)	0 (0.0)	0.024
360 days	n (%)	2 (5.0)	2 (3.4)	0 (0.0)	2 (5.4)	6 (1.6)	0.377

### 3.7 Co-prevalence of risk factors

Within the spectrum of cardiovascular risk factors, patient age, body mass index (BMI), and underlying diseases such as arterial hypertension, diabetes mellitus, chronic kidney disease, and peripheral and cerebral arterial disease were the variables independently associated with short- and long-term mortality in addition to D-dimer values (short-term: OR 1.218, CI 1.122–1.323, *p* < 0.001; long-term: OR 1.171, CI 1.095–1.252, p < 0.001). After adjusting for these variables in a multivariate model, D-dimer remained significantly associated with mortality (short-term: OR 1.201, CI 1.109–1.300, p < 0.00; long-term: OR 1.158, CI 1.079–1.242, p < 0.001). *Tables S1–S4* show details of the univariate and multivariate logistic regressions.

## 4 Discussion

### 4.1 Key findings

In this prospective, single-center cohort of unselected ED patients, D-dimer at presentation was independently associated with short- and long-term all-cause mortality, with an ROC-derived optimal cut-off of 0.75 μg/mL that performed consistently for 30- and 360-day outcomes. This threshold should be interpreted as hypothesis-generating and requires external and prospective validation before being applied in routine clinical decision-making. These associations remained significant after adjustment for age, BMI, and major cardiovascular comorbidities.

### 4.2 What is new?

Although previous studies, including the prospective analysis by Nickel et al. [6], have shown an association between increased D-dimer levels and negative outcomes in ED patients, significant gaps remain. Our study builds on these findings in three clinically relevant ways: First, we systematically measured D-dimer levels upon arrival to the ED for all included patients, thereby minimizing indication bias. Second, we assessed both short-term and long-term outcomes with follow-up up to 360 days. Third, we identified a clinically relevant subgroup of patients discharged home with elevated D-dimer levels who exhibited substantially increased one-year mortality despite being deemed stable for outpatient management. These findings complement and expand upon prior work by highlighting the potential role of D-dimer as not only a diagnostic tool but also a broadly applicable prognostic biomarker in routine ED care. [12], [13], [14], [15], [16], [17], [18], [19], [20], [23]

### 4.3 Biological plausibility

D-dimer reflects the activation of both coagulation and fibrinolysis processes, which are amplified by systemic inflammation, infection, malignancy, and cardiovascular disease. These are precisely the types of multimorbidities that drive medium-term risk in ED populations. Therefore, D-dimer may serve as an integrative marker of overall disease burden, which explains its broad prognostic utility beyond acute VTE. This concept was particularly evident during the COVID-19 pandemic when markedly elevated D-dimer levels were consistently associated with disease severity and short-term in-hospital mortality. This highlights the biomarker's role in early risk stratification for acute illnesses. Additionally, studies investigating composite biomarkers support this interpretation. For instance, the Naples Prognostic Score, which incorporates inflammatory and nutritional parameters, has been demonstrated to independently predict long-term mortality in patients with pulmonary embolism. These findings reinforce the idea that dysregulation across interconnected biological systems, rather than a single disease, drives prognosis. They also support the idea that D-dimer is a parsimonious and widely available surrogate for this global risk state. [24], [25], [26] D-dimer reflects concurrent activation of coagulation and fibrinolysis, processes amplified by systemic inflammation, infection, malignancy, and cardiovascular disease – precisely the multimorbidity profile that drives medium-term risk in ED populations. Hence, D-dimer may function as an integrative marker of overall disease burden, explaining its broad prognostic utility beyond acute VTE. Our findings are supported by recent large-scale observational data from various clinical settings. In a nationwide, population-based registry study of over 150,000 unplanned hospital visits, Brabrand et al. showed that elevated D-dimer levels at the time of admission were consistently linked to decreased survival, regardless of the underlying diagnosis or reason for admission. These results reinforce the concept of D-dimer as a marker of severity of acute illness and overall mortality risk in populations receiving emergency care. [27] Beyond the acute care setting, similar associations have been observed in the general population. In the MOLI-SANI study, Di Castelnuovo et al. found that elevated D-dimer levels were independently associated with increased all-cause mortality in a large group of seemingly healthy adults, even after adjusting for inflammatory markers. These results suggest that D-dimer reflects underlying pathophysiological processes that extend beyond overt disease states. Taken together, these findings support interpreting D-dimer as a broadly applicable biomarker of biological vulnerability that integrates signals from coagulation activation, inflammation, and chronic disease burden. In this context, our results build on previous studies by showing that this prognostic indicator can be detected at the time of ED presentation and remains relevant for short- and long-term outcomes. [18]

### 4.4 Clinical implications: “Elevated D-dimer / no VTE / discharge” pathway

In routine practice, an elevated D-dimer that does not lead to VTE confirmation is often labelled a “false positive.” Our data suggest this label is misleading: such patients carry meaningful short- and long-term risk, despite the absence of an acute thromboembolic event. A pragmatic approach could be: [1] document the elevated value (e.g., ≥ 0.75 μg/mL) in the discharge documents; [2] arrange early ambulatory follow-up (e.g., within one to two weeks) with targeted review of comorbidity and functional status; [3] consider D-dimer as part of a parsimonious prognostic panel (age, BMI, chronic kidney disease, diabetes, peripheral artery disease, etc.) to flag those who may benefit from closer monitoring or intensified chronic-care management. This preserves the diagnostic logic (no imaging cascade for VTE when pre-test probability is low and imaging was negative) while leveraging the prognostic signal for longitudinal care. [6], [20], [23], [28], [29], [30], [31], [32] Additionally, our findings underscore the potential value of D-dimer levels at the lower end of the spectrum. Patients with D-dimer levels below the conventional threshold demonstrated an excellent short- and long-term prognosis. This suggests that low D-dimer values may provide reassurance beyond ruling out VTE. For clinically stable patients, this information could support safe discharge decisions and help avoid unnecessary hospital admissions or extensive diagnostic workups. Conversely, elevated D-dimer levels should not be dismissed as mere “false positives” even in the absence of confirmed thromboembolic disease. Rather, they should prompt clinicians to consider underlying conditions, such as infection, malignancy, cardiovascular disease, or systemic inflammation. While extensive immediate diagnostics in the ED may not always be warranted, documenting and acknowledging an unexplained elevation may help ensure appropriate follow-up and further evaluation in the outpatient setting. Overall, D-dimer can serve as a bidirectional risk marker in the ED, with low values supporting clinical reassurance and discharge and elevated values identifying patients who may benefit from closer follow-up and further diagnostic clarification.

### 4.5 Avoiding unintended harm (over-testing)

A key concern is over-triggering VTE work-up purely based on an elevated D-dimer; we explicitly do not propose that. Rather, D-dimer should retain its diagnostic role for VTE per guideline-concordant pre-test assessment [11], [22], [33], and add a prognostic layer for discharge planning when VTE has been ruled out. Safeguards include: reaffirming clinical probability frameworks, pre-defining a prognostic threshold (e.g., ≥ 0.75 μg/mL), and embedding the alert in post-discharge follow-up rather than repeating or expanding ED imaging. [11], [22], [28], [29], [30], [31], [32], [33] Importantly, concerns that the broader use or interpretation of D-dimer measurements could lead to more imaging and unnecessary testing are not consistently supported by the available evidence. For example, in a study by Brabrand et al., routine measurement of D-dimer in patients with suspected SARS-CoV-2 infection did not result in a significant increase in radiological investigations. These results suggest that D-dimer testing alone does not inevitably trigger excessive diagnostic cascades when interpreted within an appropriate clinical framework.

### 4.6 Strengths and limitations

This study was conducted at a single tertiary academic center, which may limit the generalizability of our findings and introduce center-specific practice patterns. However, our study population reflects a broad, unselected spectrum of acutely ill medical patients presenting to the ED. This population encompasses a wide range of clinical conditions and comorbidities. Importantly, systematically measuring D-dimer in all included patients reduces indication bias and strengthens the internal validity of the observed associations. Together, these aspects support the external relevance of our findings, particularly in comparable, high-volume ED settings. Nevertheless, differences in healthcare systems, patient populations, and diagnostic workflows may influence the applicability of our results to other settings, especially non-tertiary or resource-limited environments. Therefore, external validation in multicenter and international cohorts is warranted. Additionally, the causes of death were not systematically adjudicated beyond broad categories and remained unclear in a significant number of cases. Although this limits conclusions regarding cause-specific mortality, it does not affect the robustness of our primary endpoint: all-cause mortality. All-cause mortality is widely considered the most objective and clinically relevant outcome measure. Although we adjusted for major comorbidities, residual confounding cannot be excluded. Moreover, even though we identified strong and independent associations between D-dimer and both short- and long-term mortality, we did not perform formal calibration or model extension analyses (e.g., D-dimer combined with age, chronic kidney disease, or diabetes) to assess incremental discrimination. Such analyses were deliberately omitted to maintain transparency and avoid overfitting in this single-center cohort. Future multicenter studies should explore whether adding D-dimer to multivariable prognostic models meaningfully improves prediction performance and clinical utility. Another limitation is that we could not systematically identify or exclude patients with confirmed VTE. Consequently, the observed association between D-dimer levels and mortality may partly reflect acute thromboembolic events. However, this also reflects real-world emergency department practice, in which D-dimer is measured before definitive diagnostic clarification. Importantly, the proportion of patients with confirmed VTE in unselected ED populations is generally low, suggesting that the observed associations are unlikely to be solely driven by thromboembolic disease. Therefore, our findings should be interpreted as reflecting D-dimer's overall prognostic value at the time of ED presentation, irrespective of the underlying cause. Future studies with systematic adjudication of VTE diagnoses should further clarify the diagnostic and prognostic value of D-dimer. Another aspect to consider is the type of D-dimer assay used. In our study, we measured D-dimer levels using a laboratory-based latex agglutination method. This method may differ from point-of-care assays, which are commonly used in some emergency or outpatient settings. Previous studies have shown that point-of-care D-dimer tests are highly sensitive, especially in low-risk patients, and can be used to rule out venous thromboembolism. [34] However, differences in assay characteristics, including calibration and specificity, may affect absolute values and cutoff thresholds. Therefore, while the overall prognostic association observed in our study is likely generalizable, the direct application of specific cutoff values (e.g., 0.75 μg/mL) to point-of-care settings should be approached with caution and requires further investigation. Although we demonstrated the good discriminatory performance of D-dimer for short- and long-term mortality, we did not perform formal calibration analyses, such as calibration plots, Brier scores, or Hosmer–Lemeshow testing. Therefore, the agreement between the predicted and observed risks cannot be fully assessed. Our primary aim was to evaluate the independent prognostic association of D-dimer and derive hypothesis-generating thresholds in a single-center cohort. Therefore, we focused on discrimination rather than optimizing model performance to avoid overfitting. Consequently, further validation of the clinical applicability of D-dimer for risk prediction is required in larger, multicenter studies, including comprehensive model development and calibration analyses.

## 5 Conclusion

In an unselected emergency department population, D-dimer levels measured upon arrival were independently associated with short- and long-term mortality. Beyond its established role in diagnosing venous thromboembolism, D-dimer may serve as a broadly available biomarker for early risk stratification, particularly for patients discharged from the ED. Incorporating D-dimer into routine risk stratification could facilitate targeted follow-up and post-discharge management. However, the proposed cutoff value (e.g., ≥0.75 μg/mL), derived from ROC analysis, should be considered exploratory and requires prospective validation before routine clinical implementation. Future multicenter studies should evaluate whether integrating D-dimer into structured risk stratification pathways improves clinical outcomes and is feasible in diverse healthcare settings.

## CRediT authorship contribution statement

**Andrea Kornfehl:** Writing – review & editing, Writing – original draft, Visualization, Validation, Methodology, Investigation, Formal analysis, Data curation, Conceptualization. **Roman Brock:** Writing – review & editing, Validation, Investigation, Data curation, Conceptualization. **Julia Oppenauer:** Writing – review & editing, Validation, Investigation, Data curation, Conceptualization. **Felix Eibensteiner:** Writing – review & editing, Validation, Investigation, Data curation, Conceptualization. **Christoph Veigl:** Writing – review & editing, Validation, Investigation, Data curation, Conceptualization. **Marco Neymayer:** Writing – review & editing, Validation, Investigation, Data curation, Conceptualization. **Karolina Valentova:** Writing – review & editing, Validation, Investigation. **Alexandra Julia Lipa:** Writing – review & editing, Validation. **Markus Müller:** Writing – review & editing, Validation. **Tim Dreier:** Writing – review & editing, Validation. **Philip Verdonck:** Writing – review & editing, Validation. **Patrick Mucher:** Writing – review & editing, Validation, Resources, Project administration, Investigation, Conceptualization. **Thomas Perkmann:** Writing – review & editing, Validation, Resources, Investigation, Conceptualization. **Helmuth Haslacher:** Writing – review & editing, Validation, Resources, Project administration, Investigation, Conceptualization. **Oliver Schlager:** Writing – review & editing, Writing – original draft, Validation, Supervision, Resources, Project administration, Methodology, Investigation, Data curation, Conceptualization. **Sebastian Schnaubelt:** Writing – review & editing, Writing – original draft, Validation, Supervision, Resources, Project administration, Methodology, Investigation, Formal analysis, Data curation, Conceptualization.

## Ethics and informed consent

Ethical approval (N°2197/2017) was provided by the Ethical Committee of the Medical University of Vienna, Austria. Patients provided written informed consent. The study complies with the Declaration of Helsinki and STROBE guidelines.

## Patient and public involvement statement

It was not appropriate or possible to involve patients or the public in the design, or conduct, or reporting, or dissemination plans of our research.

## Funding

None.

## Declaration of competing interest

None.

## Data Availability

The data underlying this article will be shared on reasonable request to the corresponding author, according to national law.
