# Supplementary material for: Short- and long-term risk stratification in acutely ill medical patients by implementing D-dimer in the emergency setting – A prospective cohort study
Source: Am Heart J Plus. 2026 Apr 13;65:100783. doi: 10.1016/j.ahjo.2026.100783 (PMC13094509; doi:10.1016/j.ahjo.2026.100783)
Supplement: Supplementary file 1 — Supplementary tables [file mmc1.docx]

**Supplements**

**Table S1.** Results of unvariate logistic regression of risk factors on short-term-mortality. OR = Odds Ratio, CI = confidence interval, LB = lower bound, UB = upper bound, µg/mL = µgramm/milliliter, BMI = body-mass-index.

| Variable | OR | CI 95% LB | CI 95% UB | p - value |
| --- | --- | --- | --- | --- |
| D-dimer (µg/mL) | 1.218 | 1.122 | 1.323 | **<0.001** |
| Age (years) | 1.082 | 1.045 | 1.122 | **<0.001** |
| Sex | 1.604 | 0.680 | 3.782 | 0.280 |
| BMI (kg/m^2^) | 0.936 | 0.864 | 1.014 | 0.160 |
| Arterial hypertension | 2.443 | 1.004 | 5.942 | **0.049** |
| Hyperlipidemia | 1.514 | 0.655 | 3.498 | 0.332 |
| Diabetes mellitus (type II) | 4.042 | 1.782 | 9.171 | **<0.001** |
| Chronic kidney disease | 4.544 | 1.833 | 11.267 | **0.001** |
| Peripheral arterial disease | 3.844 | 1.266 | 11.670 | **0.017** |
| Cerebral arterial disease | 4.954 | 1.778 | 13.800 | **0.002** |
| Coronary heart disease | 1.756 | 0.718 | 4.295 | 0.217 |
| Nicotine abuse | 0.294 | 0.621 | 0.255 | 1.511 |

**Table S2**: Results of unvariate logistic regression of risk factors on long-term-mortality. OR = Odds Ratio, CI = confidence interval, LB = lower bound, UB = upper bound, µg/mL = µgramm/milliliter, BMI = body-mass-index.

| Variable | OR | CI 95% LB | CI 95% UB | p - value |
| --- | --- | --- | --- | --- |
| D-dimer (µg/mL) | 1.171 | 1.095 | 1.252 | **<0.001** |
| Age (years) | 1.051 | 1.034 | 1.069 | **<0.001** |
| Sex | 1.598 | 0.988 | 2.585 | 0.056 |
| BMI (kg/m^2^) | 0.922 | 0.877 | 0.968 | **0.001** |
| Arterial hypertension | 1.728 | 1.080 | 2.765 | **0.023** |
| Hyperlipidemia | 1.569 | 0.976 | 2.521 | 0.063 |
| Diabetes mellitus (type II) | 1.591 | 0.934 | 2.713 | 0.088 |
| Chronic kidney disease | 3.329 | 1.850 | 5.992 | **<0.001** |
| Peripheral arterial disease | 2.227 | 1.011 | 4.904 | **0.047** |
| Cerebral arterial disease | 2.541 | 1.194 | 5.408 | **0.016** |
| Coronary heart disease | 1.548 | 0.917 | 2.615 | 0.102 |
| Nicotine abuse | 0.867 | 0.961 | 0.602 | 0.867 |

**Table S3.** Results of multivariate logistic regression of risk factors on short-term-mortality. OR = Odds Ratio, CI = confidence interval, LB = lower bound, UB = upper bound, µg/mL = µgramm/milliliter.

| Variable | OR | CI 95% LB | CI 95% UB | p - value |
| --- | --- | --- | --- | --- |
| D-dimer (µg/mL) | 1.201 | 1.109 | 1.300 | **<0.001** |
| Age (years) | 1.082 | 1.035 | 1.130 | **<0.001** |
| Arterial hypertension | 0.749 | 0.274 | 2.048 | 0.573 |
| Diabetes mellitus (type II) | 2.380 | 0.907 | 6.245 | 0.078 |
| Chronic kidney disease | 1.573 | 0.561 | 4.411 | 0.389 |
| Peripheral arterial disease | 2.267 | 0.628 | 8.187 | 0.212 |
| Cerebral arterial disease | 1.851 | 0.512 | 6.696 | 0.348 |

**Table S4.** Results of multivariate logistic regression of risk factors on long-term-mortality. OR = Odds Ratio, CI = confidence interval, LB = lower bound, UB = upper bound, µg/mL = µgramm/milliliter, BMI = body-mass-index.

| Variable | OR | CI 95% LB | CI 95% UB | p - value |
| --- | --- | --- | --- | --- |
| D-dimer (µg/mL) | 1.158 | 1.079 | 1.242 | **<0.001** |
| Age (years) | 1.040 | 1.022 | 1.060 | **<0.001** |
| BMI (kg/m^2^) | 0.890 | 0.838 | 0.945 | **<0.001** |
| Arterial hypertension | 0.945 | 0.544 | 1.642 | 0.842 |
| Chronic kidney disease | 1.764 | 0.910 | 3.419 | 0.093 |
| Peripheral arterial disease | 1.657 | 0.694 | 3.954 | 0.256 |
| Cerebral arterial disease | 1.331 | 0.542 | 3.269 | 0.532 |
